# Parental and perinatal risk factors associated with onset of IBD: a systematic literature review and meta-analysis

**DOI:** 10.3389/fgstr.2025.1621215

**Published:** 2025-10-31

**Authors:** Kristian Paul Mallon, Ciara McBride, Colm Antoine O Morain, Glen A. Doherty, Richeal Burns

**Affiliations:** ^1^ Department of Health and Nutritional Science, Atlantic Technological University, Sligo, Ireland; ^2^ The Health and Biomedical Research Centre (HEAL), Atlantic Technological University, Sligo, Ireland; ^3^ School of Medicine, Trinity College Dublin, Dublin, Ireland; ^4^ School of Medicine, University College Dublin, Dublin, Ireland

**Keywords:** inflammatory bowel disease, colitis, Crohn’s disease, parental, perinatal, risk factors

## Abstract

**Introduction:**

There is accumulating evidence that certain perinatal and prenatal factors may contribute to the onset of IBD, however evidence on some risk factors is inconsistent. The present study seeks to extend current knowledge on these risk factors and provide a comprehensive overview of which factors are associated with IBD onset and their direction of effect.

**Methods:**

A Systematic review and meta-analysis of case-control, cohort studies and randomised controlled trials (RCTs) investigating the association between parental and perinatal factors and onset of IBD was conducted. Studies were included if they reported details on patients with a diagnosis of IBD (including Crohn’s Disease [CD] and/or Ulcerative Colitis [UC]), defined and measured according to endoscopic, radiological, and histopathological findings, confirmed by a gastroenterologist or physician. Computerised bibliographic searches of Ovid MEDLINE, Web of Science, and the Cochrane Library were conducted from 01/01/2002 to the 01/01/2022. Where possible, summaries of the effects of perinatal and prenatal variables for each study were provided by calculating risk estimates using the DerSimonian and Laird random effects model. Levels of heterogeneity were evaluated using the I² statistic. Data were analysed using Stata version 17. Study protocol details are published on the International prospective register of systematic reviews (PROSPERO), registration number: CRD42022290798.

**Results:**

Fifteen eligible studies were identified, encompassing 9 case-control and 6 cohort studies, with no RCTs identified. A total of 6,507 patients with IBD were described in these studies (1,819 UC; 3,908 CD; 754 IBD; 4 IBD-unclassified patients). Three predictors of IBD risk were identified. Any poor maternal health or disease in mother during pregnancy (Pooled RR 1.78, 95% CI 1.24-2.31), maternal IBD (Pooled RR 4.59, 95% CI 1.68-7.50), and familial history (Pooled RR 2.87, 95% CI 1.80-3.93), were associated with an increased risk of overall IBD.

**Discussion:**

This systematic review and meta-analysis suggests parental and perinatal factors may have a role in the onset of IBD. These findings highlight the importance of early-life exposures for later IBD development and indicate a requirement for further research in this area.

**Systematic Review Registration:**

PROSPERO, identifier (CRD42022290798).

## 1 Introduction

Inflammatory Bowel Disease (IBD) is a chronic, debilitating, immune mediated disorder that has no cure and presents with a range of gastro-intestinal related symptoms. IBD is comprised primarily by Crohn’s disease (CD) and Ulcerative colitis (UC). The European Crohn’s and Colitis Organisation has launched the REACH strategy (1) which has places a better understanding the causes of IBD as one if it’s five strategic goals in the coming years.

Several risk factors have been associated with the development of IBD, including prenatal and early life exposures occurring during gestation and up to the end of an infant’s first year of life. Perinatal factors linked to the subsequent development of IBD include parental/biological and environmental exposures. Previous research has highlighted associations between maternal health during pregnancy (2, 3), breastfeeding (4–6), birth delivery method, family history of IBD (2), birth weight (3, 7), maternal smoking (8), antibiotic exposure (9) and IBD onset in later life. It is postulated that exposure to such factors impacts upon immune system development, leading to alterations in immune responses, and increased susceptibility to disease (10).

However, there is a paucity of evidence evaluating this association systematically whilst focussing specifically on the perinatal period. Thus, the purpose of this review is to determine where evidence exists that parental and perinatal factors are associated with the onset of IBD and to highlight the gaps in evidence to inform future studies.

## 2 Materials and methods

### 2.1 Aim and rationale

The overall aim of this review is to assess which parental and perinatal factors are associated with the onset of IBD, and to assess their direction of effect.

### 2.2 Study strategy and selection criteria

This review was developed according to the Preferred Reporting Items for Systematic Reviews and Meta-Analyses (PRISMA) guidelines. Study protocol details have been published on the International prospective register of systematic reviews (PROSPERO), registration number: CRD42022290798.

Electronic databases (Ovid MEDLINE, Web of Science, Cochrane Library) were systematically searched from 01/01/2002 to the 01/01/2022. Searches were restricted to this date range to ensure the inclusion of the most recent and relevant literature, focusing on contemporary evidence that accurately reflects the current state of knowledge and practice. Searches were restricted to English language only and human participants. We initially conducted a comprehensive search using keywords and terms related to “Inflammatory Bowel Disease,” and associated risk factors, categorized into parental and perinatal, dietary, environmental, pharmacological, microbiological, and psychosocial domains. Applying Boolean operators (“AND,” “OR”), we combined terms including “Inflammatory Bowel Disease,” “Crohn’s disease,” “Ulcerative Colitis,” “Perinatal”, “Parental”, “Diet,” “Nutrition,” “Environment,” “Environmental,” “Microbiological,” “Pharmacological,” “Psychosocial,” “Risk,” and “Risk factors.” Due to the substantial number of search results, we further refined the focus of our meta-analysis to studies evaluating parental and perinatal factors, as detailed below.

### 2.3 Study selection and data extraction

Titles and/or abstracts of studies retrieved using the search strategy and those from additional sources were screened independently by two review authors (KM and RB) to identify studies that potentially meet the inclusion criteria.

The full text of these potentially eligible studies was retrieved and independently assessed for eligibility by two review team members (KM and RB). Any disagreement between reviewers regarding eligibility of studies was resolved through discussion.

Data extracted included: author, year of publication, location and study design, sample size, method of case and control recruitment, method of parental/perinatal factor evaluation, diagnosis/reporting method, number of patients/controls, variables adjusted for, and RRs and 95% confidence intervals (CIs). Data were extracted using standardised forms and cross-checked by both reviewers.

### 2.4 Eligibility criteria

Case-control, cohort studies and randomised control trials (RCTs) which reported exposure to parental and perinatal factors; breastfeeding, caesarean section (C-section), disease during pregnancy, maternal IBD, familial IBD, low birth weight, and mother’s age were included if they reported adjusted estimates of relative risk (RR) (including odds ratios and hazard ratios) and 95% (CIs). The diagnosis of IBD (including CD and/or UC) was defined and measured according to endoscopic, radiological, and histopathological findings, confirmed by a gastroenterologist or physician.

We excluded abstracts without full texts, single case reports, review articles, animal studies and non-English language studies. Review articles were excluded as primary literature from original research articles was only considered.

### 2.5 Statistical analyses

Where possible, we provided summaries of the effects of perinatal and prenatal variables for each study by calculating risk estimates using the DerSimonian and Laird random effects model (11), to account for both within-study and between-study variation (heterogeneity).

When heterogeneity between studies was not considered high, a fixed-effect model was applied to pool the data. When comparing exposure to parental and perinatal risk factors, we compared the those who were exposed to a certain risk factor vs. those who were not exposed, in relation to onset of IBD. Levels of heterogeneity were assessed according to Higgins et al. (12), and were evaluated using the I² statistic and Q statistic. We considered heterogeneity to be low if I² statistic is 25%, moderate if I² statistic is ~50% and high if I² statistic is ~75%.

The Quality In Prognosis Studies (QUIPS) tool was applied to assess study quality and the risk of bias in the included studies (13). Where there was insufficient data to calculate pooled risk estimates, we reported the association between other parental and perinatal risk factors and IBD risk narratively. We rated the overall risk of bias in respective studies as low, medium or high. Data were analysed using Stata version 17 (Stata Corporation, College Station, TX, USA).

## 3 Results

### 3.1 Search results

**Figure 1:**
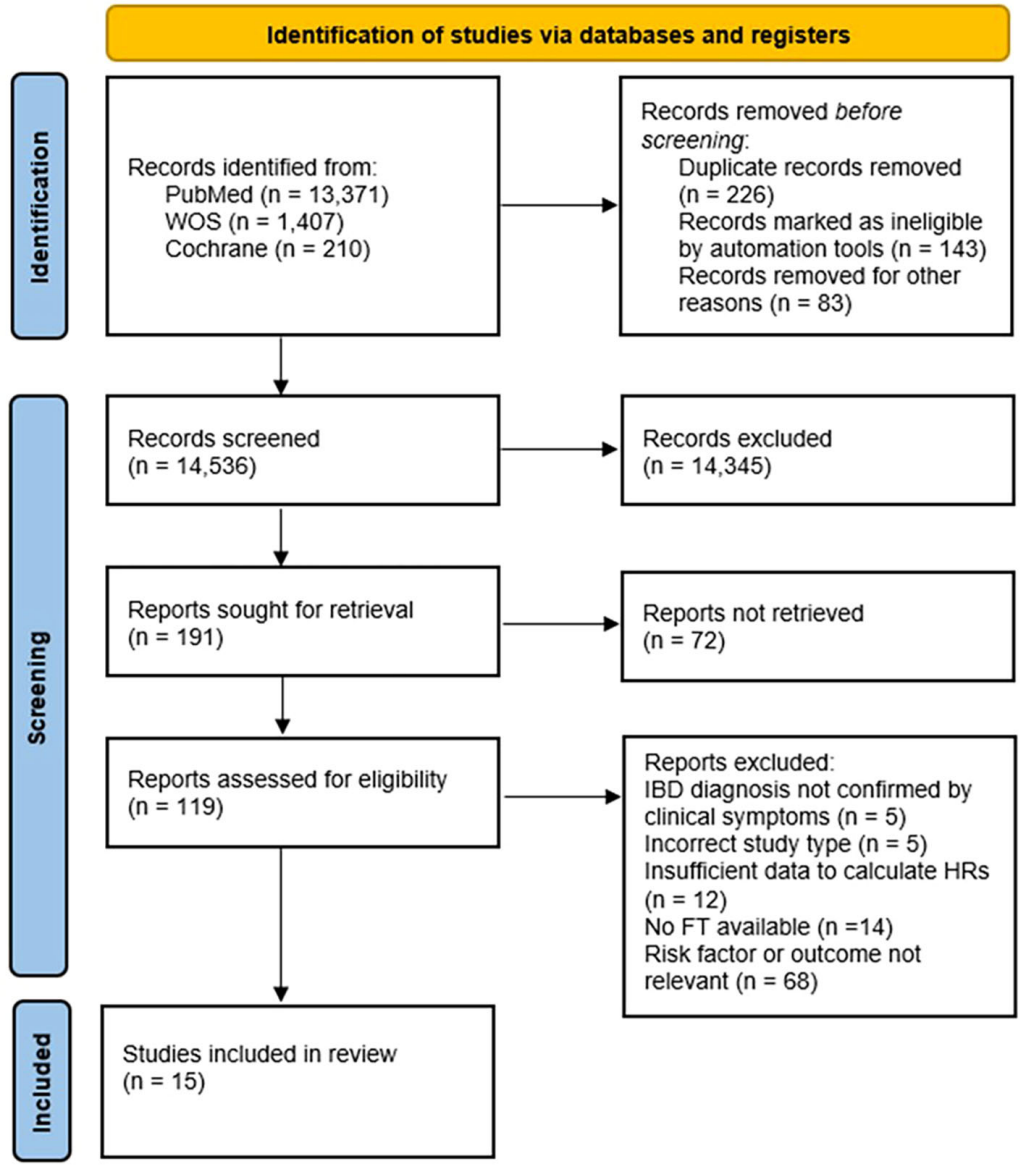
Records identified for studies relating to parental and perinatal risk factors associated with the onset of IBD.

Our initial search identified 14,809 potentially relevant articles to include, outlined in **Figure 1**. After title and abstract screening there were 119 articles that met the eligibility criteria. After full-text review and further assessment of these 119 articles. Fifteen articles were deemed suitable for study synthesis based on the eligibility criteria (**Table 1**). Case-control study design was used in 9 studies, and cohort design was used in 6 studies. No RCTs were identified. Of the 15 included studies, 10 were conducted in Europe (2, 4, 7, 8, 14–19), three in North America (3, 20, 21), one in South America (6) and one in Asia (5). A total of 6,507 patients with IBD were described in these studies (1,819 UC; 3,908 CD; 754 IBD; 4 IBDU patients).

**Table 1:** Characteristics of 15 studies investigating the association between parental and perinatal factors and IBD onset.

Study	Year	Country	Study Type	Cases	Controls	Recruitment method (recruited from)	Diagnosis/reporting method	Data collection methods/strategy	Covariates adjusted for
Baron et al. (14)	2005	France	Case-control	222 CD/60 UC	222 CD/60 UC (matched controls)	Registry data	Gastroenterologist confirmed	Questionnaire	Mother’s educational level, Father’s educational level
Bengston et al. (15)	2010	Norway	Cohort (Population based study)	24 twin pairs (at least one twin with IBD) Comparison group: 84 CD/87 UC cases	NA	Norwegian Medical Birth Registry (Twins) Norwegian population-based incidence study of IBD (The IBSEN study)	Gastroenterologist confirmed	Questionnaire	Birth weight, gestational age and sex
Boneberger et al. (16)	2011	Germany	Case-control	85 IBD	91	Gastroenterology unit of the Dr. Hauner Children’s Hospital, University Hospital of Munich	Endoscopic and radiological criteria	Questionnaire	Age, sex, high parental school education, family history of IBD, environmental factors in the first year of life (urban place of living, farm animal or pet contact), and presence of cats or dogs in the room
Burnett et al. (21)	2020	USA	Cohort	Clinical cohort: 416 IBD/262 CD/151 UC/4 IBDI Administrative cohort: 182 IBD	NA	Registry data	Gastroenterologist confirmed	Questionnaire	Maternal pre-pregnancy weight status (not overweight or obese [<25 kg/m2], overweight [25 to <30 kg/m2], obese [≥30 kg/m2]); maternal age at birth of the child, parity (0, 1, 2, or ≥3 previous live births greater than 500 g), annual income, area of residence (urban vs rural), any smoking during the pregnancy.
Du et al. (20)	2014	USA	Cohort	123 UC	NA	Registry data	Clinical, endoscopic, radiological, and histopathological criteria	Data collected and recorded in registry	Secondary ileostomy, family history of IBD, history of preoperative weight loss, age at surgery, age at UC diagnosis, history of preoperative transfusion.
Gordon et al. (4)	2022	UK	Cohort	42 CD/49 UC	NA	UK IBD Twin registry	Medical records	Questionnaire	Gastrointestinal infection prior to diagnosis, childhood illness, parental concern regarding germs, smoking history, method of delivery, infant feeding method and self-reports of diet.
Hutfless et al. (3)	2012	USA	Case-control	79 CD/92 UC	3080	Kaiser Permanente healthcare facility	Gastroenterologist confirmed	Electronic health data	Matching, sex and race (non-Hispanic white versus other), mothers age at child’s birth, Maternal infection, inpatient or outpatient visit, Gestational hypertension, pre-eclampsia, or eclampsia, Placental or amniotic problems, maternal IBD
Jakobsen et al. (17)	2013	Denmark	Case-control	59 CD/56 UC/3 IBDU	477	Local paediatric departments	Copenhagen Diagnostic criteria	Questionnaire	Age, gender, ethnicity, area of residence and socioeconomic status
Malmborg et al. (18)	2012	Sweden	Case-control	1536 CD	15,349	National Swedish patient Register	Gastroenterologist confirmed	Data collected and recorded in registry	Socioeconomic index, maternal infections during pregnancy, number of older siblings, gestational age, and mother’s age.
Roberts et al. (8)	2011	UK	Cohort	114 CD/66 UC	NA	Registry data (Oxford record linkage study)	Medical records	Data collected and recorded in registry	Year of birth, maternal age, maternal smoking
Salgado et al. (6)	2017	Brazil	Case-control	145 CD	163	Outpatient clinic	Clinical, endoscopic, radiological, and histopathological criteria	Structured interviews and questionnaire	Sex, age, race, education level, family income, rural area, housing conditions, family size in adulthood, family size in childhood, pets, breastfeeding, exposure to untreated water, vaccine (childhood), viral diseases (childhood), helminthic infections, exposure to enteric pathogens, previous appendectomy, tobacco exposure, family history.
Sonntag et al. (2)	2007	Germany	Case-control	1096 CD/763 UC	878	Hospital outpatient clinic/German Crohn’s and Colitis Foundation members	Histological assessment	Questionnaire	Disease in first year of life, disease in pregnancy, preterm birth, maternal IBD, appendectomy, ever smoking, female sex.
Strisciuglio et al. (19)	2017	Italy	Case-control	102 CD/162 UC	203	Hospital recruitment	Clinical, endoscopic, radiological, and histopathological criteria	Questionnaire	Mother’s degree, breastfeeding >3^rd^ month, father’s employment, gluten introduction <6^th^ month, no of siblings <2, autoimmune diseases, pets, bed sharing, Low adherence to Mediterranean diet, Family parasitosis
Thorsen et al. (7)	2016	Denmark	Case-control	155 CD/210 UC/19 IBDU	384	Department of Paediatrics at Hvidovre University Hospital (Denmark)	Medical records	Data collected and recorded in registry	25(OH)D, gender, ethnicity, birth weight (categorical), gestational age (categorical) and mother’s age (categorical)
Xu et al. (5)	2021	China	Cohort	71 IBD	NA	Hospital recruitment	Gastroenterologist confirmed	Questionnaire	NR

NA, Not Applicable; NR, Not reported.

### 3.2 Risk of bias assessment

Out of the 15 included studies, Risk of Bias assessment using QUIPS determined that 60% (n=9) of studies included in the analysis had a low risk of bias, 6.7% (n=1) had a moderate risk of bias and 33.3% (n=5) had a high risk of bias (**Table 2**).

**Table 2:** Summary of QUIPS scoring for the quality assessment of studies investigating the association between parental and perinatal and IBD onset.

Author (Year)	Risk of Bias Assessment						
	Study Participation	Study Attrition	Prognostic Factor Measurement	Outcome Measurement	Study Confounding	Statistical Analysis	Overall Interpretation (Risk of Bias)
Baron et al. (2005) (14)	Low	Low	Low	Low	Moderate	Low	Low
Bengtson et al. (2009) (15)	Low	Low	Low	Low	Moderate	Low	Low
Boneberger et al. (2011) (16)	Low	Low	Low	Low	Moderate	Low	Low
Burnett et al. (2020) (21)	Low	Low	Low	Low	Low	Low	Low
Du et al. (2015) (20)	Low	Low	Low	Low	High	Low	High
Gordon et al. (2022) (4)	Low	Low	Moderate	Low	Low	Low	Low
Hutfless et al. (2012) (3)	Moderate	Low	Low	Low	Low	Low	Low
Jakobsen et al. (2012) (17)	Moderate	Low	Low	Low	Moderate	Low	Moderate
Malmborg et al. (2012) (18)	Low	Low	Low	Low	Moderate	Low	Low
Roberts et al. (2011) (8)	Low	Low	Low	Low	Moderate	Low	Low
Salgado et al. (2017) (6)	Moderate	Moderate	Low	Low	High	Low	High
Sonntag et al. (2022) (2)	High	High	High	High	Moderate	Low	High
Strisciuglio et. (2017) (19)	Low	Low	Low	Low	High	Moderate	High
Thorsen et al. (2016) (7)	Low	Low	Low	Low	Moderate	Low	Low
Xu et al. (2021) (5)	Low	Low	Low	Low	High	Low	High
	Low risk of bias						
	Moderate risk of bias						
	High risk of bias						

### 3.3 Meta-analysis results

#### 3.3.1 Familial IBD history

Six studies, including four case-control (6, 16, 17, 19) and two cohort studies (15, 20), reported on the association between any family history of IBD and subsequent IBD risk. When compared with those with no family history of IBD, those with a family history of IBD had a 2.9-fold increased risk of a subsequent diagnosis of IBD in pooled analysis (Pooled RR 2.87, 95% CI 1.80-3.93), with high heterogeneity observed (I^2^ = 76.5%). Two studies (15, 19) observed the association between familial IBD and UC risk, with a RR of 2.48 (95% CI 1.27-3.69) reported in those with a family history of IBD. Another three studies (16, 17, 20) observed the association between familial IBD and IBD risk, with a RR of 6.76 (95% CI 1.34-12.18) for those with a family history of IBD (**Supplementary Figure 1**).

#### 3.3.2 Maternal IBD

Two case-control studies (2, 3) reported on the association between maternal IBD and subsequent IBD risk. Results from pooled analysis highlighted that Maternal IBD was associated with an almost 4.6-fold increased risk of a subsequent IBD diagnosis (Pooled RR 4.59, 95% CI 1.68-7.50) with no heterogeneity reported (I^2^ = 0.0%) (**Supplementary Figure 2**).

#### 3.3.3 Disease during pregnancy

Two case-control studies (2, 14) reported data on whether the pregnant mother had any other disease during pregnancy. When compared with mother’s who did not have any disease during pregnancy, in those who did have any disease during pregnancy overall risk of IBD was increased by 78% (Pooled RR 1.78, 95% CI 1.24-2.31) with no heterogeneity observed (I^2^ = 0.0%). One study (2) reported almost double the risk of CD (RR 1.97, 95% CI 1.18-2.76) in mothers reporting any other disease during pregnancy, including HELLP syndrome, hypertension, aberrant blood count and high liver enzymes (**Supplementary Figure 3**).

#### 3.3.4 C-section

Three case-control studies (2, 3, 18) and one cohort study (21) examined the association between mother having had a C-section and IBD risk. Overall, no associations were observed. Heterogeneity was low (I^2^ = 13.5%) (**Supplementary Figure 4**).

#### 3.3.5 Breastfeeding

Seven studies reported on the association between breastfeeding and IBD risk, including five case-control (2, 6, 14, 17, 19) studies and two cohort studies (4, 17). Overall, there were no significant associations observed when comparing those who were breastfed with those who were not (Pooled RR 0.95, 95% CI 0.65-1.26), with high heterogeneity observed (I^2^ = 74.5%) (**Supplementary Figure 5**).

#### 3.3.6 Low birth weight (birth weight <2,500g)

Two case-control studies (3, 7) observed the association between low birth weight (birth weight <2,500g) and subsequent IBD risk. Pooled results indicated that low birth weight was not significantly associated with onset of IBD (Pooled RR 1.38, 95% CI 0.63-2.13). No heterogeneity was observed (I^2^ = 0.0%) (**Supplementary Figure 6**).

#### 3.3.7 Mother’s age (<35 years vs. ≥35 years)

Two case-control studies (3, 7) and one cohort study (8) examined the association between mother’s age and IBD risk. Overall, no significant associations were observed when comparing mothers <35 years of age with mothers ≥35 years of age, with no heterogeneity reported (I^2^ = 0.0%) (**Supplementary Figure 7**).

#### 3.3.8 Other parental and perinatal factors

There were also a further 15 perinatal and prenatal risk factors identified which were not suitable for meta-analysis (**Table 3**). One case-control study (14) reported a 2.9-fold increased risk of CD in children who had eczema before the age of 2 years (OR 2.90, 95% CI 1.50-5.60). This study also reported that smallpox (OR 2.10, 95% CI 1.00-4.30), poliomyelitis (OR 2.60, 95% CI 1.10-6.20) and BCG vaccinations (OR 2.80, 1.00-4.30) were identified as risk factors for CD whilst MMR vaccination (OR 0.50, 95%.035-0.90) was associated with a reduction in the risk of UC. Moreover, smallpox (OR 10.0, 95% CI 1.30-208) and poliomyelitis (OR 7.0, 95% CI 1.10-151) vaccinations were also risk factors for UC in this study.

**Table 3:** Perinatal and prenatal factors not included in meta-analysis.

Perinatal/prenatal risk factor	Study	IBD subtype	Effect (95% CI)
Eczema (before the age of 2 years)	(14)	CD	2.90 (1.50-5.60)
Vaccination			
Smallpox	(14)	CD	2.10 (1.00-4.30)
Poliomyelitis		CD	2.60 (1.10-6.20)
BCG		CD	2.80 (1.00-4.30)
MMR		CD	0.50 (0.35-0.90)
Vaccination			
Smallpox	(14)	UC	10.0 (1.30-208.0)
Poliomyelitis		UC	7.0 (1.10-151.0)
High parental school education	(16)	IBD	1.63 (0.78-3.41)
Gestation (≤36 weeks vs >36 weeks)	(3)	IBD	0.60 (0.30-1.50)
		CD	0.40 (0.10-1.60)
		UC	0.60 (0.20-2.20)
Parents divorced (Yes)	(17)	IBD	1.70 (1.00-4.30)
Maternal smoking during pregnancy (Yes)	(8)	CD	2.04 (1.06-3.92)
Antibiotic prescription			
First 6 months of life	(9)	IBD	1.45 (0.80-2.62)
First 12 months of life		IBD	1.07 (0.64-1.79)
Antibiotic prescription			
First 6 months of life	(9)	CD	2.61 (1.17-5.81)
First 12 months of life		CD	1.72 (0.84-3.53)
Antibiotic prescription			
First 6 months of life	(9)	UC	0.85 (0.29-2.52)
First 12 months of life		UC	0.72 (0.28-1.86)
Family size in childhood (>7 vs 1–3 persons)	(6)	CD	1.66 (0.81-3.40)
Preterm birth	(2)	CD	1.52 (1.12-2.07)
		UC	1.38 (0.99-1.92)
Mother degree (Yes)	(19)	CD	4.80 (2.50-9.50)
Father employment	(19)	CD	1.90 (1.00-3.70)
		UC	2.30 (1.30-4.10)
Family stress	(19)	CD	1.60 (1.00-2.60)
		UC	2.20 (1.40-3.50)
Bed sharing	(19)	CD	0.30 (0.20-0.60)
No. of siblings (<2)	(19)	CD	2.10 (1.30-3.60)
		UC	2.0 (1.30-3.10)
Single (only) child (Yes)	(5)	IBD	0.30 (0.12-0.76)

One other case-control study (19) identified numerous familial risk factors for IBD, including an increased risk of CD in offspring of a mother who had previously attained a college degree (OR 4.80, 95% CI 2.50-9.50), an employed father (OR 1.90, 95% CI 1.00-3.70), family stress (OR 1.60, 95% CI 1.00-2.60) and less than two siblings (OR 2.10, 95% CI 1.30-3.60). Bed sharing exhibited a protective effect against CD (OR 0.30, 95% CI 0.20-0.60). Family stress (OR 1.60, 95% CI 1.00-2.60), an employed father (OR 2.30, 95% 1.30-4.10), and having less than two siblings (OR 2.0, 95% 1.30-3.10) also increased the risk UC. Contrastingly, a cohort study (5) reported that being a single or only child decreased the risk of IBD by up to 70% (OR 0.30, 95% CI 0.12-0.76). Other familial factors associated with IBD risk included divorced parents, with an 70% increased risk of IBD (OR 1.70, 95% CI 1.00-4.30) in offspring reported in one case-control study (17).

A nested case-control (9) study evaluating perinatal and antibiotic exposures and the risk of developing childhood-onset IBD was identified, showing a 2.6-fold increased risk of IBD (OR 2.61, 95% CI 1.17-5.81) in children who had an antibiotic prescription in their first year of life.

## 4 Discussion

This systematic review and meta-analysis of the association between parental and perinatal factors and the risk of developing IBD identified 15 studies (9 case-control and 6 cohort studies), encompassing a total of 6,507 patients with IBD (1,819 UC; 3,908 CD; 754 IBD; 4 IBDU patients).

Our review identified several links between parental and perinatal factors and IBD risk. First, we found an association between mother’s who had active disease i.e. any other comorbidity, during pregnancy and an overall pooled increased risk of IBD. We also observed that maternal IBD was associated with an almost 4.6-fold increased risk in IBD in our pooled analysis. Lastly, we found that family history of IBD was associated with an almost 6.8-fold increased risk in IBD in our pooled analysis, as well as an increased risk for UC.

Children with at least one parent with disease during pregnancy have been shown to have a 2- to 13-fold increased risk of IBD when compared with the general population (22, 23), with lifetime risk of IBD rising to up to 67% for offspring among whom both parents have IBD (24). As noted previously, pooled data from two studies in our review indicated an almost 4.6-fold increased risk for IBD in offspring when maternal IBD was present. Moreover, a 1.8-fold increased risk for offspring of mothers with disease during pregnancy was identified, when observing several other diseases in mothers during pregnancy, including, HELLP syndrome, hypertension, high blood count and high liver enzymes. It is important to note that pooled data for each respective risk factor (i.e. disease in pregnancy and maternal IBD), was calculated based on effect estimates from only two studies and thus should be interpreted with caution.

Family history of IBD was also associated with an increased risk of both IBD overall, and UC risk in our study. Family history of IBD has been highlighted as recognised risk factor for IBD onset, and has been reported in up to 8-12% of all IBD patients (25). In line with our present findings, a 2017 study reported that in 2,058 UC patients, a positive family history of IBD was reported in 31 patients, 24 (77.4%) of whom had a first-degree relative affected.

Moreover, none of the affected relatives were diagnosed with CD. Such findings suggest that genetics may play a key role in the development of IBD, possibly in conjunction with environmental factors. It has been proposed previously that interactions between genes and that environment, mediated by the innate and acquired immune system, epigenome and microbiota may help better explain the development of IBD (26). This complex interplay between genes and the environment may also help better explain our findings related to the positive association between IBD onset and mother’s disease during pregnancy and maternal IBD. The importance of genetic studies on IBD can be exemplified by the work of Hugot et al. (27), which discovered one of the first IBD-related single nucleotide polymorphisms, NOD2, which has been shown to confer susceptibility to CD. Future studies quantifying the impact of genetics in the onset of IBD, whilst controlling for potential confounding factors, will be important in developing a greater understanding of the aetiology of the disease.

Our review found no significant association between breastfeeding and IBD risk, contradicting existing evidence (28) indicating a lower incidence of paediatric-onset IBD among those who were breastfed. This earlier evidence is based on data from two earlier meta-analyses; the first including data from 17 studies published until 2003 (29), and the other including data from 7 studies published until 2009 (30). Our present review focuses solely on studies published from 2002 onward and thus may exclude many of the published studies analysed in these respective reviews. Additionally, high heterogeneity (I^2^ = 74.5%) was observed after meta-analysis of our present results observing the association between breastfeeding and IBD risk. These methodological differences may help better explain the discrepancy in findings between our present review and earlier reviews.

### 4.1 Strengths and limitations

Our study has several strengths. The likelihood for confounding may have been reduced as we extracted RRs with fully adjusted covariates. Our inclusion criteria ensured that we captured data only on IBD cases diagnosed through standardised methods including endoscopic, histological, and radiological findings (31). A risk of bias assessment was performed and cross-checked by two independent reviewers (KM and CM), which indicated that over half of the studies included in our study (60%; n=9/15) had a low risk of bias. Lastly, all data extracted for our study was cross-checked independently by two reviewers, maximising the likelihood of correct information on study characteristics and effect sizes being retrieved.

There are a several limitations to this meta-analysis. We identified no RCTs in our review, and therefore are results are informed by evidence from cohort and case-control studies, which are subject to recall bias and confounding. Additionally, for several parental and perinatal risk factors, we only had sufficient data to calculate pooled RRs from two studies, which may lead to reduced precision in overall estimates.

In summary, our results suggest that parental and perinatal factors may play an important role in the development of IBD. Additional studies of a longitudinal design with a prospective follow-up are required to determine causality. Along with the findings from our present study, such data will provide important evidence to the support the development of disease risk stratification tools, which have implications for improved efficiency and reduced health care costs associated with diagnostic delay and follow-on care. These data will have implications for the identification of key risk factors and targets for secondary prevention strategies at an early life stage, which may have the potential to alter the disease course of IBD.
