# Supplementary material for: Parental and perinatal risk factors associated with onset of IBD: a systematic literature review and meta-analysis
Source: Front Gastroenterol (Lausanne). 2025 Oct 31;4:1621215. doi: 10.3389/fgstr.2025.1621215 (PMC12952363; doi:10.3389/fgstr.2025.1621215)
Supplement: Supplementary file 1 [file Table1.docx]

**Supplementary Figure 1.** Forest plot comparing no family history of IBD vs. family history of IBD and IBD risk

**Supplementary Figure 2.** Forest plot comparing no maternal IBD vs. maternal IBD and IBD risk

**Supplementary Figure 3.** Forest plot comparing no disease during pregnancy vs. disease during pregnancy and IBD risk

**Supplementary Figure 4.** Forest plot comparing C-Section (none) vs having a C-Section and IBD risk

**Supplementary Figure 5.** Forest plot comparing not breastfed at birth vs. breastfed at birth and IBD risk

**Supplementary Figure 6.** Forest plot comparing normal birth weight (>2,500g) vs. low birth weight (<2,500g) and IBD risk

**Supplementary Figure 7.** Forest plot comparing mother’s age (<35 years vs. ≥35 years) and IBD risk
